# A Simple Pneumatic Method of Removing Foreign Substances from the Nasal Cavity of Children

**Published:** 1891-02

**Authors:** 


### A SIMPLE PNEUMATIC METHOD OF REMOVING FOREIGN SUBSTANCES FROM THE NASAL CAVITY OF CHILDREN.

Dr. T. J. Slaton, of Greenville, Ky., describes this method as follows: "The operator places a thin cloth over the child's mouth, applies his finger to the nostril not containing the substance, pressing sufficiently to close the lumen, and then puts his mouth to the child's and gives two or three strong puffs. The substance will fly out nine times out of ten. I have blown them ten feet out, often. I have used this method for the last ten years with but one failure; in that case I failed but forced the substance so near the anterior nasal opening that I had no trouble in removing it with forceps.

---

HEADACHES OF CHILDREN.—Charles L. Dana says the headaches of children can be best controlled by small doses of iodide of iron or the citrate of iron and quinine.—*Ex.*

